# Influence of Self-Compassion, Burden of BPSD, Communication Behavior, and Nursing Work Environment on Person-Centered Care for Patients with Dementia Among Long-Term Care Hospital Nurses

**DOI:** 10.3390/healthcare14010015

**Published:** 2025-12-20

**Authors:** Yong Min Kim, Mi Heui Jang, Min Jung Sun

**Affiliations:** 1 Department of Nursing, Graduate School, Kyung Hee University, Seoul 02447, Republic of Korea; hongyu98@khu.ac.kr; 2 College of Nursing Science, East-West Nursing Research Institute, Kyung Hee University, Seoul 02447, Republic of Korea; 3 Department of Nursing, Gangdong University, Eumseong-gun 27600, Republic of Korea; mjsun@gangdong.ac.kr

**Keywords:** nurse, long-term care, dementia, self-compassion, burden, communication, nursing work environment, person-centered care

## Abstract

**Objectives**: This study aimed to identify the factors influencing person-centered care (PCC) among nurses working at long-term care hospitals for patients with dementia and to propose strategies for strengthening their capacity to provide PCC. **Methods**: Guided by the ecological model, this descriptive study examined the effects of personal factors (self-compassion and the burden of behavioral and psychological symptoms of dementia [BPSD]), interpersonal factors (communication behavior), and organizational factors (nursing work environment) on PCC. Participants were 152 nurses who had worked for more than two months at four long-term care hospitals in Seoul and Gyeonggi Province, South Korea. Data were collected between 8 January and 4 February 2024, and analyzed using SPSS version 23.0. **Results**: Hierarchical multiple regression analysis showed that the strongest predictors of PCC were the nursing work environment (β = 0.36, *p* < 0.001), having received dementia-related education twice (β = 0.26, *p* = 0.008), self-compassion (β = 0.23, *p* = 0.017), having received dementia-related education three or more times (β = 0.22, *p* = 0.036), and communication behavior (β = 0.20, *p* = 0.026). The final model (Model 3) explained 41.5% of the variance in PCC (adjusted R^2^ = 0.415, F = 5.70, *p* < 0.001). **Conclusions**: To strengthen PCC among nurses in long-term care hospitals, comprehensive efforts to improve the nursing work environment are essential. Institutional support should particularly focus on securing sufficient nursing staff and ensuring adequate material resources. In addition, continuous dementia-related education and training programs that foster self-compassion and communication skills among nurses are recommended.

## 1. Introduction

Dementia is an irreversible condition that progressively impairs cognitive, behavioral, and psychological functions, eventually making it impossible for affected individuals to perform daily activities without assistance [1]. Advances in medical technology have increased life expectancy, leading to a sharp rise in the number of people with dementia, with an estimated 55 million individuals worldwide currently living with the disease [2]. This rapid growth has created a substantial global economic and social burden [3]. In Korea, the number of dementia patients is projected to rise from approximately 940,000 in 2022 to 3.15 million by 2050 [4]. To address this challenge, the Korean government introduced the “National Responsibility for Dementia Policy” in 2017. Under this policy, patients with mild dementia receive services through in-home care, community dementia relief centers, and long-term care facilities, while patients with severe dementia are primarily treated in long-term care hospitals [5]. Despite this expansion of dementia care services, cultural perceptions of dementia continue to pose challenges. In both Asian and Western societies, the term “dementia” often carries negative connotations, leading to significant social stigma for both patients and their families [6,7], and this stigma is particularly pronounced in Asian countries where cultural attitudes strongly influence care experiences and quality of life [8].

Person-centered care (PCC) is a holistic and exemplary approach to dementia care that views individuals with dementia as persons with unique life histories, seeks to uphold and strengthen their personhood, and emphasizes respect for their lives [9,10]. The concept of personhood was initially introduced by Tom Kitwood [11] to explain the essential humanity of individuals living with dementia, emphasizing that personhood involves treating them as fundamentally human—regardless of age or cognitive ability—and maintaining feelings of trust, safety, and well-being as core outcomes of PCC. Previous studies have shown that PCC for dementia patients can reduce medication use, behavioral and psychological symptoms, and depression while improving emotional well-being and overall quality of life [12,13,14]. Furthermore, practicing PCC has been found to benefit caregivers as well, lowering job stress and tension while increasing both personal and professional satisfaction and enhancing confidence in their caregiving abilities [15].

Previous conceptual analyses of PCC indicate that the healthcare environment—both physical and cultural—can influence how PCC is delivered in practice [16]. In Korea, dementia care has been influenced by a family-centered caregiving culture grounded in Confucian values, which contrasts with Western approaches that prioritize autonomy and individual decision-making [17]. A recent scoping review further suggests that cultural context may influence the feasibility of PCC implementation, particularly in Asian settings where dementia-related stigma and task-oriented care remain prevalent [18]. While such cultural characteristics provide an important contextual backdrop for understanding PCC in Korea, examining how PCC is practiced in daily nursing care requires a framework that extends beyond cultural factors. In this regard, the ecological model features interpersonal and organizational factors—highlighting the structural and environmental conditions within clinical practice settings without reducing PCC to individual attributes. Therefore, this study investigates how nurses in Korean long-term care hospitals provide PCC for patients with dementia within these ecological layers of care.

Self-compassion refers to treating oneself with kindness and understanding rather than self-criticism, particularly in difficult circumstances [19]. Nurses with high levels of self-compassion tend to extend the same tolerance to others, which helps them better understand their patients and more effectively meet each patient’s unique needs [20,21]. Self-compassion has also been reported to decrease emotional exhaustion and contribute positively to personal growth [22]. These findings suggest that self-compassion plays an important role in promoting nurses’ inner development and enhancing their emotional resilience.

Behavioral and psychological symptoms of dementia (BPSD), including hallucinations, anxiety, agitation, and sleep disturbances, place a substantial physical and psychological burden on caregivers [23,24]. These symptoms arise from multiple causes and manifest differently depending on each patient’s individual characteristics [25]. Therefore, identifying the underlying causes of a patient’s symptoms and providing individualized management strategies are essential. In this context, person-centered care can play a crucial role by delivering tailored nursing interventions that alleviate BPSD and reduce the burden on caregivers, including nurses.

Due to cognitive decline, individuals with dementia often experience communication difficulties, which may lead to withdrawal from social interactions, emotional isolation, and a diminished quality of life [26]. Communication with dementia patients is central to preserving their personhood. When caregivers use disrespectful communication, patients may respond with negative behaviors such as discomfort, aggression, or resistance [27]. Accordingly, nurses must engage in respectful and nonjudgmental communication with patients with dementia. Improving nurses’ communication skills enables them to better identify patients’ individual needs, listen attentively, respect autonomy, and express empathy—factors that are expected to play a pivotal role in the successful implementation of person-centered care.

The nursing work environment refers to organizational characteristics that support nurses in delivering professional care to patients [28]. A positive nursing work environment has been recognized as a significant factor in enhancing perceived quality of care and promoting person-centered care [15,29]. However, in long-term care hospitals, nurses are often responsible not only for administering medications and providing basic care but also for numerous non-medical tasks, as many patients are unable to perform activities of daily living independently. These additional tasks include assisting with feeding, changing diapers and clothing, and managing personal items such as snacks and supplies [30,31]. Furthermore, because of staff shortages, nurses frequently take on the additional responsibility of training and supervising nursing assistants. This increased workload intensity creates significant challenges to the effective practice of person-centered care [32]. Therefore, it is necessary to closely examine the nursing work environment in long-term care hospitals and assess its impact on the delivery of person-centered care.

Although person-centered care (PCC) provided by nurses in long-term care hospitals is critically important for patients with dementia, previous studies have lacked a comprehensive approach that accounts for diverse influencing factors. Specifically, prior research tended to focus primarily on individual psychological characteristics, with limited studies examining interpersonal and organizational factors [33,34]. To address this gap, the present study, based on the ecological model [35], sought to comprehensively investigate the effects of personal factors (self-compassion and the burden of BPSD), interpersonal factors (communication behavior), and organizational factors (nursing work environment) on person-centered care, drawing on prior research.

## 2. Materials and Methods

### 2.1. Study Design, Participants, and Procedures

This descriptive survey study, grounded in the ecological model [35], examined factors influencing person-centered care (PCC) among nurses in long-term care hospitals. Participants were registered nurses with at least two months of clinical experience caring for patients with dementia, recruited from four long-term care hospitals with capacities of over 200 beds in Seoul and Gyeonggi Province. Hospitals were selected through convenience sampling based on geographic accessibility.

Prior to data collection, institutional approval was obtained from each participating hospital. Participants were recruited with the cooperation of the nursing departments, which assisted in distributing the questionnaires to eligible nurses. Nurses in managerial or administrative roles, or those not involved in direct patient care, were excluded.

The required sample size was calculated using the G*Power 3.1 program with a significance level of 0.05, statistical power of 80%, and a medium effect size of 0.15, indicating a minimum of 150 participants. Considering a 10% dropout rate, 170 paper-based questionnaires were distributed. Eligible nurses voluntarily completed the questionnaires and returned them in sealed envelopes to ensure anonymity.

All measurement instruments used in this study were validated Korean versions with established reliability and validity, and permission for use was obtained from the original authors. After removing 18 questionnaires with missing key items, data from 152 participants were analyzed.

All participants were informed of the study objective and procedures, and participation was voluntary. A small gift card was provided as appreciation. Data collection was conducted over a single four-week period from 8 January to 4 February 2024.

### 2.2. Measurement

Self-Compassion: This study used the Korean version of the Self-Compassion Scale (K-SCS) adapted and validated by Kim [36] from Neff’s original Self-Compassion Scale (SCS) [37]. The instrument consists of 26 items divided into six subscales: three positive (self-kindness, common humanity, mindfulness) and three negative (self-judgment, isolation, over-identification). Items are rated on a 5-point Likert scale. Negatively worded items were reverse-scored. Consistent with recent recommendations, self-compassion was evaluated using the total score rather than separate subscale scores. Cronbach’s α was 0.92 in Neff’s original study [37] and 0.86 in the present study.

Burden of Behavioral and Psychological Symptoms of Dementia (BPSD): This study used a tool originally developed and validated by Kim [38] for assessing behavioral and psychological symptoms of dementia in older residents of long-term care facilities, and later revised and supplemented by Kim [39]. The tool consists of 25 items across six subscales: aggressive behavior (6 items), agitated behavior (6 items), resistance to care (5 items), physical symptoms (3 items), neurological symptoms (3 items), and psychiatric symptoms (2 items). The frequency of symptoms is rated on a 4-point scale (0 = none, 1 = rarely, 2 = sometimes, 3 = always). For nurses who had observed such symptoms, the associated burden was rated on a 4-point Likert scale (0 = not burdensome to 3 = very burdensome). Higher scores indicate greater caregiver burden. Cronbach’s α was 0.91 in Kim’s study [39] and 0.94 in the present study.

Communication Behavior: Communication behavior with dementia patients was measured using the tool developed and validated by Lee & Gang [40]. The scale consists of 18 items across four subscales: conversation response management (5 items), relationship regulation (3 items), emotional expression (6 items), and understanding enhancement (4 items). Each item is rated on a 5-point Likert scale ranging from 1 (strongly disagree) to 5 (strongly agree). Higher scores indicate more frequent use of effective communication behaviors and strategies. Cronbach’s α was 0.88 in the original study [40] and 0.85 in the present study.

Nursing Work Environment: The nursing work environment was assessed using the Korean version of the Practice Environment Scale of the Nursing Work Index (PES-NWI), originally developed by Lake [28] and adapted and validated for use in Korea by Cho [41]. The scale includes 29 items across five subscales: nurse participation in hospital affairs (9 items), foundations for quality of care (9 items), nurse manager ability, leadership, and support (4 items), staffing and resource adequacy (4 items), and collegial nurse–physician relations (3 items). Each item is rated on a 4-point Likert scale from 1 (strongly disagree) to 4 (strongly agree). Higher scores reflect a more positive perception of the nursing practice environment. Cronbach’s α was 0.82 in Lake’s original study [28] and 0.93 in the present study.

Person-Centered Care: Person-centered care (PCC) was measured using the Korean version of the Person-Centered Practice Inventory—Staff (PCPI-S), originally developed by Slater et al. [42] and translated and validated by Kim & Tak [43]. The Korean version of the PCPI-S reflects three of the four domains proposed by McCormack & McCance [9] and consists of 51 items representing 16 elements: 5 prerequisites, 7 items for the care environment, and 4 items for the care processes. Each item is rated on a 5-point Likert scale ranging from 1 (strongly disagree) to 5 (strongly agree). Higher scores indicate higher levels of person-centered care provision. Cronbach’s α was 0.95 in Kim & Tak’s study [43], and 0.96 in the present study.

### 2.3. Ethical Approval

This study was approved by the Institutional Review Board of Kyung Hee University (KHSIRB-23-466). All participants provided written informed consent, and ethical principles—including voluntary participation, confidentiality, and anonymity—were strictly observed.

### 2.4. Statistical Analysis

The data collected in this study were analyzed using SPSS version 23.0. Descriptive statistics—including frequency, percentage, mean, and standard deviation—were used to examine participants’ general characteristics, self-compassion, burden of BPSD, communication behavior, nursing work environment, and levels of person-centered care. To assess differences in person-centered care across participant characteristics, independent t-tests, one-way analysis of variance, and Scheffé post hoc tests were performed. Prior to conducting the main analyses, the normality of the study variables was assessed using the Shapiro–Wilk test. Pearson’s correlation coefficients were calculated to examine the relationships among self-compassion, burden of BPSD, communication behavior, nursing work environment, and person-centered care. Finally, hierarchical multiple regression analysis was conducted to identify the factors influencing person-centered care, with only the general characteristics that demonstrated statistically significant differences in the univariate analyses being entered as independent variables.

## 3. Results

### 3.1. Difference in PCC According to the General Characteristics of Participants

Differences in person-centered care according to participants’ general characteristics are presented in Table 1. Statistically significant differences were observed for age (F = 4.14, *p* = 0.018), marital status (t = 2.94, *p* = 0.004), job position (F = 4.32, *p* = 0.015), years of experience at the current long-term care hospital (F = 3.42, *p* = 0.019), number of dementia-related education sessions (F = 6.26, *p* = 0.002), and dementia care practicum experience (t = 2.19, *p* = 0.030).

### 3.2. Correlations Among Self-Compassion, Burden of BPSD, Communication Behavior, Nusing Work Environment

The correlations among self-compassion, burden of BPSD, communication behavior, nursing work environment, and person-centered care are presented in Table 2. Person-centered care was significantly and positively correlated with self-compassion (r = 0.38, *p* < 0.001), communication behavior (r = 0.40, *p* < 0.001), and the nursing work environment (r = 0.40, *p* < 0.001).

### 3.3. Factors Influencing PCC in Participants

Hierarchical multiple regression analysis was conducted to identify the factors influencing person-centered care among nurses, based on the ecological model. Variables that showed significant differences in person-centered care by general characteristics were entered as dummy variables. Guided by the ecological model, predictors were entered sequentially by theoretical level—individual-level factors (self-compassion and burden of BPSD), interpersonal-level factors (communication behavior), and organizational-level factors (nursing work environment)—to examine their incremental explanatory power. Results are summarized in Table 3.

Prior to conducting hierarchical multiple regression analysis, basic assumptions were tested. The Durbin–Watson statistic for autocorrelation of the dependent variable was 1.61, which is close to 2, indicating that the assumption of independence of residuals was satisfied. Variance inflation factor (VIF) values ranged from 1.11 to 3.15, all below 10, confirming the absence of multicollinearity.

Model 1 included general characteristics (age, marital status, job position, years of experience at the current long-term care hospital, number of dementia-related education sessions, and dementia practicum experience) as well as individual factors (self-compassion and burden of BPSD). Significant predictors were receiving two dementia-related education sessions (β = 0.19, *p* = 0.030), receiving three or more dementia-related education sessions (β = 0.17, *p* = 0.048) and self-compassion (β = 0.37, *p* < 0.001). The model was significant (F = 4.14, *p* < 0.001) and explained 22.5% of the variance.

Model 2 added the interpersonal factor, communication behavior. Significant predictors included receiving two dementia-related education sessions (β = 0.19, *p* = 0.019), receiving three or more dementia-related education sessions (β = 0.17, *p* = 0.042), self-compassion (β = 0.31, *p* < 0.001), and communication behavior (β = 0.33, *p* < 0.001). The model was significant (F = 4.01, *p* < 0.001) and explained 31.5% of the variance, an increase of 9%.

Model 3 added the organizational factor, nursing work environment. Significant predictors were receiving two dementia-related education sessions (β = 0.19, *p* = 0.010), self-compassion (β = 0.30, *p* < 0.001), communication behavior (β = 0.30, *p* < 0.001), and nursing work environment (β = 0.34, *p* < 0.001). The final model was significant (F = 7.93, *p* < 0.001) and explained 42.3% of the variance, an increase of 10.8%.

## 4. Discussion

This study, grounded in the ecological model [35], found that self-compassion, communication behavior, and the nursing work environment were positively correlated with person-centered care. Hierarchical multiple regression analysis further revealed that the factors influencing person-centered care among nurses in long-term care hospitals caring for patients with dementia were, in order of influence, the nursing work environment, the number of dementia-related education sessions, self-compassion, and communication behavior. Together, these variables explained 42.3% of the variance in person-centered care. Viewed through an ecological model, this pattern of findings suggests that person-centered care is shaped by factors operating across multiple levels, including individual, interpersonal, and organizational contexts, underscoring the importance of multi-level approaches rather than interventions focused solely on individual competencies. Because this study used a cross-sectional design, these relationships should be interpreted as associations rather than causal effects.

In this study, the nursing work environment emerged as the most influential factor affecting person-centered care. This finding supports previous research showing that the work environment in long-term care facilities significantly impacts person-centered care practices among nurses and staff [44,45]. In other words, the more positively nurses perceive their work environment, the more likely they are to implement person-centered care. Therefore, multifaceted efforts at the institutional level are needed to improve the nursing work environment in long-term care hospitals. Among the subdomains of the nursing work environment, the lowest scores were reported for nurse staffing and material support, which reflects the reality of current conditions in long-term care hospitals. In Korea, a differential inpatient fee system has been in place since 2008, classifying healthcare institutions into five levels based on the nurse-to-patient ratio. Institutions are provided with additional reimbursement according to their level to encourage quality improvement. However, in long-term care hospitals, up to two-thirds of the total nursing workforce may be substituted with nursing assistants [46]. This policy incentivizes facilities to hire lower-cost nursing assistants instead of registered nurses, thereby redistributing professional responsibilities to less-trained staff. Consequently, nurses face increased burdens of supervising unlicensed personnel, which contributes to job dissatisfaction, reluctance to work in long-term care hospitals, and higher turnover rates. This cycle perpetuates a persistent shortage of nurses [47]. Such shortages not only negatively affect the nursing work environment but also pose a serious barrier to establishing a person-centered care environment. Although care assistants may provide limited support in basic caregiving tasks, person-centered care for patients with dementia fundamentally requires the professional judgment and relational competencies of registered nurses. Therefore, the roles of nurses and care assistants should be clearly differentiated, and nurses should maintain leadership in providing and coordinating dementia care. Developing strategies to optimize teamwork while preserving the essential role of nurses will be crucial for enhancing PCC in long-term care settings. To foster a more positive nursing work environment in long-term care hospitals, legal and policy-level improvements are essential, including stricter limitations on replacing nurses with assistants and revising staffing standards. These changes should be accompanied by active institutional support and initiatives.

Another factor influencing person-centered care identified in this study was the number of dementia-related education sessions. Nurses who had received such education two or more times demonstrated significantly higher levels of person-centered care. This finding aligns with prior studies indicating that nurses with dementia-related education are more aware of and committed to person-centered care than those without such training [44,48]. In addition, it has been reported that nurses with dementia education experience perceive a lower burden of dementia-related care compared to those without training [49], which may further support their ability to implement person-centered practices. However, some studies, such as Lee & Jung [50], reported no significant association between dementia care knowledge and person-centered care. In this study, dementia- or PCC-related education refers to non-standardized training, the format and content of which vary across institutions. Therefore, differences in educational exposure among nurses may influence the effectiveness of PCC implementation. Thus, to strengthen the generalizability of this study’s findings, further research with repeated and systematic designs is warranted.

The third factor influencing person-centered care in this study was self-compassion. This finding supports previous research showing that higher levels of self-compassion are associated with a greater ability to effectively address the individual needs of patients [21]. Self-compassion has also been shown to reduce negative thinking [51] and decrease avoidant tendencies [52], which may enable nurses to move beyond negative cognitive patterns, accept patients with dementia as they are, and provide care tailored to each patient’s unique needs. Compassion, by definition, involves recognizing the suffering of others, responding emotionally, and being motivated to alleviate that suffering—one of the core values of person-centered care [53]. A positive correlation between self-compassion and caregiving behaviors has also been observed [54], suggesting that compassion is a critical component in delivering authentic person-centered care [55]. Furthermore, self-compassion can be cultivated through personal training and has been shown to positively influence surrounding individuals [56]. Accordingly, within the context of long-term care hospitals, it is necessary to design and implement structured educational programs and practice-based interventions to enhance nurses’ self-compassion.

This study also revealed that communication behavior among nurses caring for patients with dementia in long-term care hospitals significantly influenced person-centered care. However, few studies have examined the relationship between communication behavior and person-centered care in this population using the same measurement tools, making direct comparisons challenging. Nevertheless, prior studies with nurses caring for cancer patients in general hospitals [57] and for patients experiencing cancer-related pain [58] also reported that communication behavior significantly affects person-centered care, consistent with the findings of the present study. For nurses working with dementia patients, the ability to recognize and respond to patient discomfort through both verbal and nonverbal communication is essential. Such communication behaviors are likely to have positively contributed to person-centered care in this study. Moreover, this finding is supported by the theoretical framework proposed by McCormack & McCance [9], which highlights communication ability as one of the core elements of the person-centered nursing process [58].

However, the burden of BPSD did not show a significant association with PCC in the present study. One possible explanation is that most previous research has examined PCC primarily as an intervention strategy to reduce BPSD, rather than exploring whether the perceived burden of BPSD itself influences nurses’ ability to deliver PCC [33]. In addition, recent literature has reported that person-centered care plans and non-pharmacological approaches can reduce the use of PRN antipsychotic medications for managing BPSD—particularly when sufficient staffing levels and educational opportunities are available [59]. This suggests that institutional support and adequate training may buffer the impact of BPSD-related burden on caregiving behaviors, potentially diminishing its direct effect on PCC. Therefore, BPSD burden might not emerge as a significant predictor of PCC when nurses have access to educational resources, structured teamwork, or opportunities for professional adaptation. Future research should investigate whether psychological resilience, communication skills, or organizational resources act as mediators or moderators in the relationship between BPSD burden and PCC among dementia care nurses.

Although this study focused on PCC from the perspective of nurses, future research should also evaluate PCC in relation to patient outcomes and the experiences of service users and cares. Determining whether communication behaviors and other nursing competencies translate into improvements in patient well-being, family satisfaction, and care burden would provide a more comprehensive understanding of the real-world impact of PCC in dementia care.

The present findings further support the ecological model by demonstrating that PCC is shaped by multiple contextual levels rather than by individual factors alone. At the personal level, self-compassion helped nurses maintain emotional resilience and recognize the unique needs of patients. At the interpersonal level, communication behavior underscored that PCC requires relational engagement through empathy, listening, and respectful interaction. At the organizational level, both the nursing work environment and access to dementia-related education reflected institutional capacity to support PCC. These findings suggest that PCC is not simply an individual competency but a contextual practice that emerges when personal resources, relational dynamics, and structural support are aligned within the care environment.

International studies have similarly emphasized that PCC is shaped by multiple contextual levels. Organizational support and staffing have been shown to strongly influence PCC [60], while interpersonal factors such as communication and empathy are essential for implementing PCC in dementia care. In addition, a recent qualitative meta-synthesis reported that nurses’ personal competencies, relational engagement, and institutional resources must align to enable PCC in hospital settings [61]. These findings are consistent with the ecological perspective adopted in the present study and support the interpretation of PCC as a multidimensional practice rather than an individual skill.

In conclusion, this study confirmed that the nursing work environment, self-compassion, and communication behavior significantly influence person-centered care among nurses caring for patients with dementia in long-term care hospitals. These findings demonstrate the importance of these factors and provide a foundation for developing strategies to strengthen person-centered care competencies in this nursing population.

This study has several limitations. First, it involved a convenience sample of nurses from four long-term care hospitals in Seoul and Gyeonggi Province, and the single-country context of Korea may limit the generalizability of the findings to other regions or healthcare systems. In addition, data were collected during a single four-week period, which may not capture temporal variation in clinical practice. The cross-sectional design also prevents causal interpretations of observed relationships. Finally, because all variables were assessed using self-report questionnaires, response bias may have influenced the results. Future research should include diverse institutions across multiple countries, employ probabilistic sampling, and utilize longitudinal or mixed-method designs to enhance external validity and deepen understanding of the mechanisms underlying person-centered care.

## 5. Conclusions

This study identified the nursing practice environment, frequency of dementia-related training, self-compassion, and communication behavior as the key factors influencing person-centered care (PCC) among nurses caring for patients with dementia in long-term care hospitals. Based on these results, strengthening the supportive work environment, providing regular dementia-focused education, and fostering self-compassion and communication competencies are essential for improving PCC. In addition, policy-level efforts to enhance staffing and reimbursement standards are needed to ensure the sustainable delivery of PCC in long-term care settings.

## Figures and Tables

**Table 1:** Difference in PCC according to general characteristics (*N* = 152).

Variables	Categories	*n* (%)	Person-Centered Care		
			M ± SD	t/F(*p*) Scheffe	Cohens’ d
Gender	Female	127 (83.6)	184.81 ± 21.47	0.08 (0.933)	0.02
	Male	25 (16.4)	184.40 ± 27.12		
Age (years)	20~39 ^a^	64 (42.1)	178.75 ± 21.62	4.14 (0.018) * b > a	0.47
	40~59 ^b^	58 (38.2)	189.36 ± 24.69		
	≥60 ^c^	30 (19.7)	188.60 ± 16.13		
Marital status	Married	89 (58.6)	189.12 ± 22.81	2.94 (0.004) **	0.49
	Unmarried	63 (41.4)	178.56 ± 20.41		
Religion	Yes	69 (45.4)	185.70 ± 20.45	0.48 (0.634)	0.08
	No	83 (54.6)	183.95 ± 23.98		
Education	College	55 (36.2)	184.15 ± 23.90	2.60 (0.078)	0.38
	University	83 (54.6)	182.99 ± 22.00		
	≥Master	14 (9.2)	197.50 ± 14.21		
Position	Staff Nurse ^a^	119 (78.3)	182.25 ± 23.34	4.32 (0.015) * c > a	0.48
	Charge Nurse ^b^	11 (7.2)	187.00 ± 16.49		
	≥Head Nurse ^c^	22 (14.5)	197.09 ± 14.76		
Total working experience (years)	<1	14 (9.2)	190.57 ± 22.86	1.87 (0.137)	0.39
	1–3	16 (0.5)	179.13 ± 16.88		
	3–5	10 (6.6)	172.00 ± 19.92		
	≥5	112 (73.7)	185.96 ± 22.39		
Working experience in long-term care facility (years)	<1 ^a^	40 (26.3)	184.15 ± 22.81	3.42 (0.019) * d > c	0.53
	1–3 ^b^	39 (25.7)	178.54 ± 17.72		
	3–5 ^c^	15 (9.9)	177.50 ± 20.93		
	≥5 ^d^	58 (38.2)	191.44 ± 23.91		
Work shift	Three shifts	51 (33.6)	181.08 ± 24.69	1.63 (0.186)	0.36
	Two shifts	50 (32.9)	184.34 ± 18.78		
	Fixed Night shifts	18 (11.8)	183.17 ± 27.76		
	Regular work	33 (21.7)	191.88 ± 19.72		
Experience of dementia education	No	45 (29.6)	180.60 ± 21.07	1.49 (0.140)	0.27
	Yes	107 (70.4)	186.48 ± 22.80		
Number of dementia-related education experiences	1 time ^a^	61 (40.1)	177.31 ± 23.75	6.26 (0.002) ** b, c > a	0.58
	2 times ^b^	51 (33.6)	188.27 ± 19.93		
	≥3 times ^c^	40 (26.3)	191.58 ± 20.36		
Practical experience with dementia patients	Yes	60 (39.5)	189.62 ± 23.24	2.19 (0.030) *	0.36
	No	92 (60.5)	181.57 ± 21.36		
Experience living with family members with dementia	Yes	26 (17.1)	189.04 ± 22.63	1.08 (0.284)	0.023
	No	126 (82.9)	183.86 ± 22.33		

* *p* < 0.05, ** *p* < 0.01, M ± SD: Mean ± Standard Deviation. Lowercase letters a, b, c and d denote groups for the Scheffe test.

**Table 2:** Correlations among the research variables.

Variable	M ± SD	1	2	3	4	5	6
1. Self-compassion	90.02 ± 12.51	1					
2. Frequency of BPSD experiences	28.78 ± 13.46	−0.04 (0.664)	1				
3. Burden of BPSD	22.66 ± 13.79	0.08 (0.336)	0.63 (<0.001) **	1			
4. Communication behavior	69.04 ± 7.17	0.11 (0.173)	0.19 (0.022) *	0.00 (0.991)	1		
5. Nursing practice Environment	77.83 ± 11.48	0.01 (0.909)	0.01 (0.905)	−0.08 (0.360)	0.14 (0.085)	1	
6. Person-centered care	184.64 ± 22.51	0.38 (<0.001) **	−0.01 (0.906)	−0.04 (0.632)	0.40 (<0.001) **	0.40 (<0.001) **	1

* *p* < 0.05, ** *p* < 0.01, M ± SD: Mean ± Standard Deviation; BPSD: behavioral and psychological symptoms of dementia.

**Table 3:** Hierarchical multiple regression of significant predictors of person-centered care.

	Model 1			Model 2			Model 3			VIF
	B	β	*p*	B	β	*p*	B	β	*p*	
Constant	119.74		<0.001	61.75		0.001	17.28		0.360	
Age (years) (40–59)	−3.18	−0.07	0.570	3.26	0.07	0.551	6.27	0.14	0.215	3.15
Age (years) (≥60)	−5.37	−0.10	0.403	0.40	0.01	0.948	4.04	0.07	0.479	2.70
Marital status (married)	7.28	0.16	0.185	2.81	0.06	0.593	−0.55	−0.01	0.910	3.00
Position (charge Nurse)	1.46	0.02	0.828	0.55	0.01	0.930	1.78	0.02	0.760	1.19
Position (≥head Nurse)	6.89	0.11	0.204	2.82	0.04	0.586	4.21	0.07	0.377	1.47
Working experience in long-term care facility (years) (1–3)	−4.62	−0.09	0.318	−3.86	−0.08	0.375	−2.55	−0.05	0.524	1.59
Working experience in long-term care facility (years) (3–5)	−9.05	−0.12	0.162	−7.87	−0.11	0.196	−5.49	−0.08	0.327	1.54
Working experience in long-term care facility (years) (≥5)	1.00	0.02	0.840	−0.63	0.01	0.892	0.64	0.01	0.882	2.24
Number of dementia-related education experiences (2 times)	8.87	0.19	0.030	9.03	0.19	0.019	9.11	0.19	0.010	1.42
Number of dementia-related education experiences (≥3 times)	8.80	0.17	0.048	8.50	0.17	0.042	6.08	0.12	0.115	1.50
Practical experience with dementia patients (Yes)	6.66	0.15	0.062	5.18	0.11	0.124	3.23	0.07	0.299	1.20
Self-compassion	0.65	0.37	<0.001	0.55	0.31	<0.001	0.53	0.30	<0.001	1.25
Frequency of BPSD experiences	0.02	0.01	0.917	−0.13	−0.08	0.410	−0.15	−0.09	0.289	1.90
Burden of BPSD	−0.16	−0.10	0.295	−0.06	−0.04	0.657	−0.01	−0.01	0.954	1.84
Communication behavior				1.08	0.33	<0.001	1.00	0.30	<0.001	1.24
Nursing practice environment							0.65	0.34	<0.001	1.11
F(*p*) R^2^(adj R^2^)	4.14 (<0.001) 0.297 (0.225)			5.63 (<0.001) 0.383 (0.315)			7.93 (<0.001) 0.484 (0.423)			

Durbin–Watson’s auto-correlation coefficient (upper critical limit). Reference: unmarried, staff nurse, total working experience (<1), experience of dementia education (1 time), practical experience for dementia patients (no).

## Data Availability

The raw data supporting the conclusions of this article will be made available by the authors upon reasonable request after signing a confidentiality agreement.
